# Parental Perceptions of Giardiasis: A Study in an Outpatient Paediatric Hospital Setting in Havana, Cuba

**DOI:** 10.5402/2013/364647

**Published:** 2012-12-03

**Authors:** Pedro Almirall, Angel A. Escobedo, Yohana Salazar, Maydel Alfonso, Ivonne Ávila, Sergio Cimerman, Isabel V. Dawkins

**Affiliations:** ^1^Department of Epidemiology, Municipal Centre of Hygiene, Epidemiology and Microbiology, Calle 8 No. 406 Esquina a 19, Vedado, 10400 La Habana, Cuba; ^2^Department of Gastro-enterology, Nutrition and Parasitology, Academic Paediatric Hospital “Pedro Borrás”, Calle F No. 616 Esquina 27, Vedado, 10400 La Habana, Cuba; ^3^Department of Child Health, National Institute of Hygiene, Epidemiology and Microbiology, Calle Infanta No. 1158 Esquina a Llinas, Cerro, 10300 La Habana, Cuba; ^4^Department of Child Health, Faculty of Medicine “Comandante Manuel Fajardo”, Calle D esquina a Zapata, Vedado, 10400 La Habana, Cuba; ^5^Gastro-intestinal unit, Department of Paediatrics, Academic Paediatric Hospital “Centro Habana”, Benjumeda y Morales, Cerro, 10600 La Habana, Cuba; ^6^Department of Infectious diseases, Institute of Infectious Diseases “Emilio Ribas”, Rua Zacarias de Gois, 966/41 São Paulo, SP, Brazil

## Abstract

*Background*. *Giardia lamblia* is an important cause of diarrhoeal disease throughout the world. Giardiasis— a mild and self-limiting disease that this protozoan causes— is perceived as a harmful disease. *Aim.* To explore the general level of awareness about giardiasis, clinical features, mode of transmission, prevention, and consequences and describe the sources and channels of information caregivers would prefer using to be informed about this disease. *Methods*. A cross-sectional survey was conducted among caregivers attending to the outpatient paediatric hospital setting in Havana. *Results*. A total of 202 caregivers were interviewed. Nearly 73% considered giardiasis as a modern problem, and 39% considered that it could be a fatal disease. Although 76.7% were aware that small intestine is the organ affected, other localizations were cited. Abdominal pain and diarrhoea were recognized as the commonest symptoms. Around one-third could identify that giardiasis may spread through drinking unboiled water and unwashed vegetables other incorrect ways were mentioned; respondents with more than 12 years of formal education were more likely to have better knowledge. *Discussion*. Strategies to control giardiasis need to be through an integrated approach aiming at boosting caregivers' knowledge and encouraging healthcare workers to act as a readily available source for health information.

## 1. Introduction

*Giardia lamblia*, probably the most frequent pathogenic intestinal protozoan infection in man, is an important cause of diarrhoeal disease in children and adults throughout the world. Although giardiasis—the disease this protozoan causes—is usually perceived as mild and self-limiting, symptoms generally subside within 2-3 weeks in otherwise healthy individuals. This infectious disease may have both immediate and long-term consequences including chronic diarrhoea with or without dehydration and intestinal malabsorption, recurrent abdominal pain, and weight loss [1]. Additionally, it has been currently related to chronic fatigue [2], postinfectious irritable bowel syndrome [3, 4], and particularly, in early childhood, poor cognitive function and failure to thrive [5]; all of these have attracted an increasing attention to this protozoan infection in the recent years. 

Despite its worldwide distribution, *Giardia* is more common in developing countries. Data from surveys, excluding documented outbreaks, indicate that in industrialized countries the prevalence rate is between 2% and 5%. In contrast, it ranges from 20% to 30% in developing countries [1]. Data on the prevalence of giardiasis in Cuba is limited; in a national survey the overall prevalence of *Giardia* infection was estimated to be 6.02% [6]. However, higher prevalences have been found among young children attending day-care centres and primary schools [7–10]. Clinical giardiasis also gives the impression to be a common reason for hospitalization in paediatric hospitals in the capital of Cuba [11, 12]; where, according to a study of risk factors for *Giardia* infection among hospitalized children, it seems that, at least at the individual level, giardiasis-prevention activities in Havana should be focused on health education to improve personal hygiene and food-related practices [12]. 

Health education (HE) continues to be one of the most important strategies in the fight against intestinal parasites [13]. Appropriate management of giardiasis, including patient education to promote adherence to preventive measures that may protect against infection and reinfection, not only might reduce the frequency of outpatient clinic visits and hospitalizations for giardiasis, but also could reduce the costs associated with this disease. 

In childhood giardiasis, mothers and other caregivers, are therefore, of foremost importance in recognizing the disease and seeking treatment for their wards. Very little is known about the perceptions of parents and carers when dealing with giardiasis in the paediatric setting. Their ideas and behaviours concerning giardiasis might enhance or interfere with the effectiveness of giardiasis prevention and control. Understanding their perceptions concerning this disease is an important step towards the disease control. A qualitative study in Cuba by Escobedo et al., in 2011, reported deficiencies in the children caregivers' knowledge and practices concerning giardiasis [14]. 

It is important to assess the current knowledge about giardiasis before devising an intervention strategy for education campaigns among general population. The purpose of this study was to (1) explore the perceptions of a group of caregivers about medical aspects of giardiasis (clinical features, modes of transmission, prevention, and consequences), its prevention and treatment; (2) assess the sources and channels of information caregivers would prefer using to be informed about giardiasis. All of this might highlight areas where the support of health care professionals can be improved. 

## 2. Study Population and Methods

### 2.1. Study Design and Setting

This was a descriptive cross-sectional study conducted between January and March 2010, at Academic Paediatric Hospital Centro Habana (APHCH), Havana, Cuba. This 226-bed study hospital is a government-funded and public facility which provides secondary and tertiary medical care and active ambulatory and emergency services, within all clinical and surgical specialities, for children from all over Cuba but mainly to the paediatric population from the municipalities of Centro Habana, Cerro, Habana Vieja, and Plaza municipalities.

### 2.2. Inclusion and Exclusion Criteria

The study included respondents aged >18 years who were willing to participate in the study and gave individual verbal consent. Only health professionals and students in the medical professions were excluded to avoid selection bias.

### 2.3. Questionnaire

A structured questionnaire was designed on the basis of a qualitative research conducted in the same hospital by the authors [14]. The questionnaire was peer-reviewed and discussed with health care professionals working within paediatric wards and also was pilot-tested on random patients' relatives at the hospital over a 1-week period to evaluate the language used, to verify the relevance of the questions, and for clarity and consistency. Results were retested after completion to verify the answers. The questionnaire was then modified accordingly. It focused on basic demographic data (age and gender), education, and questions about their knowledge regarding giardiasis, mode of transmission, organ affected, its suggestive signs and symptoms, prevention, most reliable method of diagnosis, and whether giardiasis was a curable disease. Additionally, caregivers were also assessed about from which sources and channels they would like to receive information on giardiasis. It took around 10 minutes to complete an interview. Answers were categorized into either correct (if matching the medically correct answer) or incorrect.

### 2.4. Questionnaire Administration

All consecutive parents/carers who fulfilled the eligibility criteria for inclusion and who agreed to participate were recruited if they were either new attenders or reattending. Respondents were adult members of patients' families (parents/carers) who visited the outpatient clinic of APHCH for gastrointestinal problems. After the interviewers, who were three of the research team authors (Y. Salazar, M. Alfonso, and I. V. Dawkins), explained the purpose of the study and obtained individual verbal consent from the respondents, they administered the questionnaire to parents/carers, while waiting for the consultation of their child. The interviews were conducted in private to maintain confidentiality and to reduce the influence of relations of peers.

### 2.5. Ethical Approval

The study was approved by the APHPC's Ethics for Research Committee. Verbal informed consent was obtained from the study participants before the research instruments were administered. Confidentiality of all information obtained from participants was maintained by not allowing information to be accessible to nonmembers of the research team.

### 2.6. Data Analysis

Database was prepared using Epi Info Version 6.0, and accordingly data entry was done. Frequency distributions were obtained. In the exploration of possible associations between respondent characteristics and awareness about transmission and prevention, analysis was done taking into account education of respondents, which was categorized into two categories (less than 12 years of formal education or ≥12 years of formal education) and age of respondents (less than 40 year of age and ≥40 years of age). Crude odds ratio (OR), their 95% confidence intervals (CIs) and *P* values were calculated with *α* set at 0.05.

## 3. Results

### 3.1. Sociodemographic Characteristics

The response rate of this study was 100%. Of the 202 caregivers interviewed, 177 (87.6%) were females and 25 (12.4%) were males. The mean was 35.9 years (range 18–70). A substantial proportion of caregivers, 157 (77.7%), had received 12 years of formal education or more, and 128 (63.3%) were employed outside home.

### 3.2. General Knowledge about Giardiasis Characteristics

Of the 202 caregivers, more than half (72.7%) considered that giardiasis is a modern problem, and, although almost half (47.5%) stated that this disease could not lead to death, 39.1% did, and 13.4% did not know. Small intestine was correctly identified as the site of giardiasis by 125 (61.9%) of the caregivers. However, more than one organ was mentioned as a possible target of *Giardia* infection including gallbladder (66 (32.7%)) and liver (54 (26.7%)). For 127 (62.9%) of respondents those who harbour *Giardia* infection not necessarily feel or know about it. Thirty-two (15.8%) of the respondents did not have knowledge about the signs and symptoms of giardiasis. Those having knowledge mentioned abdominal pain (77.6%), diarrhoea (70.6%), and loss of weight 59.4% as the symptoms of giardiasis. However, other clinical manifestations including hives, itching, spots in the skin, and tiredness were mentioned for more than 30% (Table 1).

### 3.3. Diagnostic Tests

The majority of caregivers, 171 (84.6%), considered the microscopy of duodenal aspirate as the most effective diagnostic tool for giardiasis. The microscopy of faecal smears was only cited by 18 (8.9%) respondents (Table 1).

### 3.4. Transmission

One hundred and sixty-nine caregivers (83.6%) in this study knew that *Giardia* could be transmitted through drinking unboiled water and unwashed vegetables and fruits intake. The other modes of transmission mentioned by interviewees were walking barefoot and using utensils previously used by patients with giardiasis (Table 2). Those with an education of 12 years of formal education or more were four times more likely to be aware of prevention than those with less than 12 years of education (OR = 4.5, 95% CI = 1.9–10.8). Interaction between knowledge of transmission and age was tested and found to have no significance (Table 3).

### 3.5. Treatment and Prevention

Nearly all caregivers (95.5%) were aware that giardiasis was a curable disease (Table 4). Almost all the respondents 175 (86.3%) considered giardiasis to be preventable, most of them (169 (83.6%)) believe that prevention could be achieved by drinking boiled water, washing hands, and washing vegetables and fruits; 86.3%, 89.6%, and 91.5%, respectively. Other actions including avoiding walking barefoot and avoiding utensils used by patients with giardiasis were also mentioned by a group of them (Table 5). Those with an education of 12 years of formal education or more were nearly three times more likely to be aware of prevention than those with less than 12 years of education (OR = 2.8, 95% CI = 1.3–5.9). No significant association was observed between knowledge of prevention and age (Table 5).

### 3.6. Sources and Channels

One hundred and fifty-nine of the caregivers stated that they did not receive information on giardiasis during the last year from any source or channel. Caregivers were asked which methods they would prefer to know or to improve their knowledge about giardiasis. Among the suggested sources and channels, television was mentioned by 130 (64.3%), nurses and medical doctors 107 (52.9%), printed materials—leaflets, posters, and pamphlets—94 (46.5%), and radio 35 (17.3%).

## 4. Discussion

The goals of treating symptomatic giardiasis properly are to minimize the duration of illness, ensure cure, stop transmission of infection, and could also be to prevent the emergence of drug resistance. Caregivers' input, as in other areas of medicine involving children, seems to be critical to clinical management in paediatric giardiasis. The importance of providing caregivers with appropriate information is to enable them to participate effectively in giardiasis recognition, and preventative measures should be recognized.

Even when there were some important gaps in various issues, it was encouraging to observe that in most issues caregivers' perceptions on giardiasis closely paralleled that of the biomedical understanding. This coincidence should be used as an entry point for a broader HE activity, which is then expanded to also accommodate other disease aspects, that is, its mode of transmission, signs and symptoms, and appropriate prevention methods, to provide accurate and complete knowledge on giardiasis.

Internationally, this disease is gaining increasing attention, first as a reemerging infectious disease in industrialized countries and, second, as a part of the “Neglected Disease Initiative” by the World Health Organization [15]. Its diagnosis has been established progressively with improving sanitation and with fading of the role of fatal epidemic infections as the overwhelming everyday problem. These, together with the fact that in Cuba high prevalences have been reported among young children attending day-care centres and primary schools [7, 8], might be justifying the perception held by most respondents that this disease is a modern problem. Additionally, *Giardia* has also been frequently isolated from faeces of Cuban children with diarrhoea, and it seems to be a common cause of children hospitalization [11, 12, 16]. These could have led some caregivers to conclude that this disease could cause death.

*Giardia* organisms primarily affect the proximal small intestine; however, during the last decades, there have been various reports concerning other localizations of *Giardia* trophozoites including gallbladder [17], urinary tract [18], gastric mucosa [19–21], colonic and ileal mucosa [22, 23], and pancreas [24]. The majority of caregivers were able to recognize that *Giardia* infection is frequently asymptomatic, and diarrhoea and abdominal pain are the clinical manifestations of symptomatic giardiasis. This is probably due to the educational message in the mass media stating that two of the primary clinical features of symptomatic intestinal parasitic diseases are diarrhoea and abdominal pain. This could be of importance, taking into account that an early treatment—in some aspects—could depend upon prompt recognition of symptoms and signs of giardiasis in the household, mainly by caregivers. Other clinical manifestation very frequently mentioned by the caregivers was urticaria. It was surprising because, although this sign has been reported associated with giardiasis in the scientific literature [25–28], it is not so common. Considering the frequent asymptomatic nature of this infection and the high prevalence of this protozoan worldwide, urticaria most of the times indicates that it is necessary to look for an alternative diagnosis. Nevertheless, it should be stated that, coincidently, in a survey of knowledge, perceptions and practices about giardiasis in 63 gastroenterologist of Havana, it was evidenced that 55.6% of these specialists in Havana stated that this parasitic infection was a frequent cause of cutaneous manifestations [29]. 

The intermittent excretion of *Giardia* cysts might be affecting the perceptions of the microscopic study of faecal specimens which for most of the caregivers considered useless. At the same time, there was also an overreliance on the microscopic examination of duodenal aspiration. This could be a reflection of a common practice in the clinical setting. Two studies on knowledge and practices on giardiasis among gastroenterologist and dermatologists in Havana showed that 52.4% of gastroenterologists [29] and 64% of dermatologist [30] considered that duodenal aspiration was the best way to diagnose this parasitic disease. Certainly, duodenal aspirate is a valuable diagnostic aid for avoiding delays in diagnosis and prolongation of patient morbidity. However, this situation could be counterproductive because other parasitologic diagnoses—potentially causing disease—including intestinal helminths and protozoa could be lost.

The caregivers demonstrate a general knowledge of giardiasis transmission and prevention. However, there were some important gaps concerning these two issues which indicate that people today tend to combine all of the general preventive measures against intestinal parasitic infections, maybe, as a reflection of a result of previous activities directed at intestinal parasites control. Additionally, there exists a link between hygiene and infection, which is a common theme in the lay literature, and caregivers could be able to make connections and draw inferences from previous similar experiences. *G. lamblia* remains the leading waterborne diarrhoea-causing disease [31, 32], either when people drink accidentally or swallow water from contaminated or untreated sources (no heat inactivation, filtration, or chemical disinfection). Outbreaks of giardiasis linked to drinking water and food handlers have been documented. The correct treatment of water and food and hand hygiene (i.e., hand washing with soap and water or the use of a waterless, alcohol-based hand rub) are considered the most important measures in preventing infections transmitted via the faecal-oral route. It has been recently shown in a systematic review the impact of interventions to improve hand washing with soap in the community could reduce diarrhoea risk by 47% [33]. In the USA, an outbreak of giardiasis, involving multiple modes of transmission, eventually stopped, possibly influenced by a vigorous hand washing campaign [34]. 

In the present study, the higher educational level of the caregivers had a positive influence on the knowledge of transmission and prevention of giardiasis. The parents' educational level is one of the factors of health inequalities among children [35]. Education is important for health because of the opportunities it creates to obtain better material living conditions; well-educated people are less likely to be unemployed, and more likely to have full-time jobs, fulfilling work, and high income. A good example could be a study carried out in Peru where participants with greater wealth indexes were associated with protection against *Giardia* and persistent *Giardia* infections (>14 days) [36]. In a study carried out in Portugal, children with mothers without any education were more likely to be infected with *Giardia* than those with educated mothers. Similarly, children living with fathers with no education had a 12.26 times higher risk of being infected with *Giardia* than those with educated fathers [37]. This highlights the need for giardiasis HE programmes and material targeted at groups with special needs. Even in a country like Cuba, where the educational level is very high, groups still exist where low education and thus higher risk of diseases, such as giardiasis, may interact to create an unfavourable situation. 

To target populations for prevention messages, it is important to know which sources and channels of information are most frequently reported as being used to keep informed about a specific topic. Consistent with a previous study [11], it was found that television was the preferred channel of information, followed closely behind by their medical doctors, and printed materials. 

HE materials such as posters and brochures may be an effective way to disseminate health information, providing such information in the local language that could be displayed at prominent places, such as health facilities and community centres. At present, access to culturally appropriate and easy-to-understand educational materials on giardiasis is lacking. Based on this study, it may be recommended that HE materials oriented towards increasing the knowledge in different issues of giardiasis should be developed. An interesting experience comes from a study carried out in schools in south Tehran. The case group and their mothers were separately covered by the HE programmes that included washing hands with soap, cutting nails, washing vegetables, and making the families knowledgeable about giardiasis and its effects on their children. All the programmes were performed by HE methods, that is, poster, video film, face-to-face meetings, and pamphlets. The control group had not undergone any health education programme. Three months later, a triple stool examination was done in both groups to check reinfection. After the implementation of the programmes, the mean of the mother's awareness about controlling of giardiasis increased from 6.54% to 27.16% (*P* = 0.00001), without any variation in the control group in this item. The mean of children's information about giardiasis and its methods of control in the case and control groups before implementation of the HE programmes was 4.76% and 4.86%, respectively, but after the implementation of the programs, it increased to 16.3% in the case group but remained at 4.86% in the control group (*P* = 0.00001). After antigiardial treatment, both groups were cured of the *Giardia* infection. After implementation of HE programs, the rate was 23.3% in the case group but 86.7% in the control group, indicating a significant difference between the two groups [38].

The interpersonal communication, which is the most effective method of communicating health information [39], that takes place in the doctor's offices and the waiting room may offer the opportunity for physicians and other health care professionals to display their potential to play a significant role in promoting individual patient action and contributing to the development of social norms concerning health behaviour. Health care workers (medical doctors and nurses) occupy a position of high credibility and trust which make their pronouncement well received; they have a well-recognized role encouraging prevention. However, as key communicators, they need the necessary skills to carry out activities that allow discussions, feedback, and explanation to the target audience. In the Cuban context, some studies carried out among gastroenterologist, and dermatologists have evidenced the necessity of improving the giardiasis management and prevention information they provide to their patients [29, 30]. This might be a fruitful area for future health intervention campaigns. 

## 5. Limitations of the Study

Study participants were restricted to caregivers who were recruited from only one health facility, and, therefore, their views may be biased by contact with the same paediatric gastroenterology team and their standard educational practices. Participants were recruited among parents/carers attending to the hospital (a majority resided in Havana), and thus we likely failed to capture the views of people in other provinces. As parents were recruited from those taking their children to the outpatient clinic for gastroenterology and within population are likely to be a large group of children who have giardiasis, it is reasonable to assume that parents of these children are more likely to know what giardiasis is than parents from the general population. Additionally, it should be noted that people attending health facilities at a hospital are expected to have a relatively greater awareness about common health issues. There is not a previous study in this area, and hence this questionnaire survey was the first to explore some of the issues in detail. Face-to-face administration of the questionnaire might have led to some degree of bias in the interviewees' responses.

## 6. Conclusions

The study has revealed reasonable knowledge of the symptoms of giardiasis; however, there is a need for educational intervention directed towards correcting misconceptions. There was a need for an effective education programme focusing on giardiasis transmission and prevention which should be emphasised in order to avoid reinfections. The central necessity of caregivers' involvement in developing educational strategies that reflect their understandings and interpretations of this disease should not be overlooked.

## Figures and Tables

**Table 1:** Responses to questions on communicable nature, mode of transmission, possibility of cure, and knowledge about symptoms of giardiasis among 202 caregivers.

Response to questions	No.	(%)
Is it a modern problem?		
Yes	147	(72.7)
No	45	(22.3)
Do not know	10	(5.0)
May lead to death		
Yes	79	(39.1)
No	96	(47.5)
Do not know	27	(13.4)
The organ affected by *Giardia *		
Small intestine	125	(61.9)
Gallbladder	66	(32.7)
Liver	54	(26.7)
Stomach	11	(5.4)
Lungs	1	(0.5)
Bones	1	(0.5)
Colon	1	(0.5)
Do not know	39	(19.3)
If one is infected by *Giardia*, one will		
Not necessary feel or know about it	127	(62.9)
Not have symptoms	0	(—)
Have symptoms	39	(19.3)
Do not know	36	(17.8)
Symptoms and signs of giardiasis		
Abdominal pain^a^	132	(65.3)
Diarrhoea^a^	120	(54.4)
Loss of weight^a^	101	(50)
Hives^a^	88	(43.5)
Itching^a^	66	(32.6)
Spots in the skin^a^	55	(27.2)
Tiredness^a^	23	(11.3)
Fever^a^	16	(7.9)
Blood in faeces^a^	12	(5.9)
Do not know	32	(15.8)
Most effective diagnostic tool		
Microscopy of duodenal aspirate	171	(84.6)
Microscopy of faeces	18	(8.9)
Biopsy	2	(0.9)
Do not know	9	(4.4)
Is giardiasis preventable?		
Yes	175	(86.6)
No	19	(9.4)
Do not know	8	(4)
Is giardiasis curable?		
Yes	191	(94.5)
No	9	(4.4)
Do not know	2	(0.9)

^a^Percentages are of the persons that knew at least one symptom.

**Table 2:** Correctly answered questions about the transmission of giardiasis among the 202 caregivers who participated.

Answered correctly that giardiasis is transmitted	*n* = 202	
	No.	(%)
Through drinking unboiled water (yes)	189	(93.5)
Through unwashed vegetables and fruit intake (yes)	176	(87.1)
Through walking barefoot (no)	145	(71.7)
Through utensils used by patients with giardiasis (no)	101	(50)
During delivery (no)	185	(91.5)
Through drinking unboiled water; unwashed vegetables and fruit intake	169	(83.6)
Score of correct answers		
All 5 correct answers	63	(31.1)
Any 4 correct answers	81	(40.1)
Any 3 correct answers	47	(23.2)
Any 2 correct answers	8	(3.9)
Only 1 correct answer	0	(—)
No correct answer	0	(—)
Answer “I do not know” to all	3	(1.5)

**Table 3:** Factors associated with the knowledge that giardiasis is transmitted by drinking unboiled water and unwashed vegetables and fruit intake.

	Investigated	At least 1 incorrect answer		All 2 correct answers		OR^a^ (95% CI^b^)
		(*n* = 33)		(*n* = 169)		
Age						
Under 40 years	125	22	(17.6)	103	(82.4)	1
40 years and over	77	11	(14.3)	66	(85.7)	1.3 (0.6–3.0)
Educational level						
Below high school	45	16	(35.5)	29	(64.4)	1
High school and above	157	17	(10.8)	140	(89.2)	4.5 (1.9–10.8)

^a^Odds Ratio. ^b^Confidence interval.

**Table 4:** Perceived methods of prevention reported by caregivers.

Answered correctly that giardiasis is prevented	*n* = 202	
	No.	(%)
Feeding boiled water (yes)	175	(86.3)
Hand washing before feeding (yes)	181	(89.6)
Washing fruits and vegetables (yes)	185	(91.5)
Avoiding walking barefoot (no)	136	(67.3)
Avoiding utensils used by patients with giardiasis (no)	135	(66.8)
Through drinking boiled water, washing hands before		
eating, and washing vegetables and fruit	150	(74.2)
Score of correct answers		
All 5 correct answers	74	(36.6)
Any 4 correct answers	72	(35.6)
Any 3 correct answers	51	(25.2)
Any 2 correct answers	1	(0.5)
Only 1 correct answer	2	(0.9)
No correct answer	2	(0.9)

**Table 5:** Factors associated with the knowledge that giardiasis may be prevented by drinking boiled water, washing hands before eating, and washing vegetables and fruit.

	Investigated	At least 1 incorrect answer		All 3 correct answers		OR^a^ (95% CI^b^)
		(*n* = 52)		(*n* = 150)		
Characteristic						
Age						
Under 40 years	125	36	(28.8)	89	(71.2)	1
40 years and over	77	16	(20.8)	61	(79.2)	1.5 (0.8–3.2)
Educational level						
Below high school	45	19	(42.2)	26	(57.8)	1
High school and above	157	33	(21.0)	124	(79 )	2.8 (1.3–5.9)

^a^Odds Ratio. ^b^Confidence interval.

## References

[1] Escobedo AA, Almirall P, Robertson LJ (2010). Giardiasis: the ever-present threat of a neglected disease. *Infectious Disorders - Drug Targets*.

[2] Mørch K, Hanevik K, Rortveit G (2009). Severity of *Giardia* infection associated with post-infectious fatigue and abdominal symptoms two years after. *BMC Infectious Diseases*.

[3] Hanevik K, Dizdar V, Langeland N, Hausken T (2009). Development of functional gastrointestinal disorders after *Giardia lamblia* infection. *BMC Gastroenterology*.

[4] Wensaas K-A, Langeland N, Hanevik K, Mørch K, Eide GE, Rortveit G (2012). Irritable bowel syndrome and chronic fatigue 3 years after acute giardiasis: historic cohort study. *Gut*.

[5] Berkman DS, Lescano AG, Gilman RH, Lopez SL, Black MM (2002). Effects of stunting, diarrhoeal disease, and parasitic infection during infancy on cognition in late childhood: a follow-up study. *The Lancet*.

[6] Rojas L, Núñez FA, Aguiar PH (2012). Segunda encuesta nacional de infecciones parasitarias intestinales en Cuba, 2009. *Revista Cubana de Medicina Tropical*.

[7] Núñez FA, Hernández M, Finlay CM (1999). Longitudinal study of giardiasis in three day care centres of Havana City. *Acta Tropica*.

[8] Arencibia AA, Escobedo AA, Núñez FA, Almirall P (2001). Parásitos intestinales en niños que asisten a una escuela primaria urbana de Ciudad de la Habana. *Boletín Epidemiológico del Instituto Pedro Kourí*.

[9] Mendoza D, Núñez FA, Escobedo A (2001). Parasitosis Intestinales en 4 círculos infantiles de San Miguel del Padrón, Ciudad de la Habana, 1998. *Revista Cubana de Medicina Tropical*.

[10] Escobedo AA, Caflete R, Nuñez FA (2008). Prevalence, risk factors and clinical features associated with intestinal parasitic infections in children from San Juan y Martínez, Pinar del Río, Cuba. *West Indian Medical Journal*.

[11] Escobedo AA, Almirall P, Alfonso M (2011). Hospitalization of Cuban children for giardiasis: a retrospective study in a paediatric hospital in Havana. *Annals of Tropical Medicine and Parasitology*.

[12] Bello J, Núñez FA, González OM, Fernández R, Almirall P, Escobedo AA (2011). Risk factors for *Giardia* infection among hospitalized children in Cuba. *Annals of Tropical Medicine and Parasitology*.

[13] Pereira AP, Alencar MF, Cohen SC (2012). The influence of health education on the prevalence of intestinal parasites in a low-income community of Campos dos Goytacazes, Rio de Janeiro State, Brazil. *Parasitology*.

[14] Escobedo AA, Almirall P, Alfonso M (2011). Caregiver perspectives for the prevention, diagnosis and treatment of childhood giardiasis in Havana City, Cuba. A qualitative study. *Acta Tropica*.

[15] Savioli L, Smith H, Thompson A (2006). *Giardia* and *Cryptosporidium* join the “neglected diseases initiative”. *Trends in Parasitology*.

[16] Núñez FA, González OM, Bravo JR, Escobedo AA, Gonzaléz I (2003). Intestinal parasitosis in children admitted to the Pediatric Teaching Hospital of Cerro, Havana City, Cuba. *Revista Cubana de Medicina Tropical*.

[17] Goldstein F, Thornton JJ, Szydlowski T (1978). Biliary tract dysfunction in giardiasis. *American Journal of Digestive Diseases*.

[18] Meyers JD, Kuharic HA, Holmes KK (1977). *Giardia lamblia* infection in homosexual men. *British Journal of Venereal Diseases*.

[19] Reynaert H, Fernandes E, Bourgain C, Smekens L, Devis G (1995). Proton-pump inhibition and gastric giardiasis: a causal or casual association?. *Journal of Gastroenterology*.

[20] Widgren S (2001). *Giardia lambua* associated gastrits. Case report. *Revue Medicale de la Suisse Romande*.

[21] Oberhuber G, Stolte M, Bethke B, Ritter M, Eidt H (1993). Gastric giardiasis: analysis of biopsy specimens from 191 patients infected with *Giardia lamblia*. *European Journal of Gastroenterology and Hepatology*.

[22] Oberhuber G, Kastner N, Stolte M (1997). Giardiasis: a histologic analysis of 567 cases. *Scandinavian Journal of Gastroenterology*.

[23] Heagley DE, Jakate S (2012). Giardiasis confined to the terminal ileum. *Clinical Gastroenterology and Hepatology*.

[24] Nakano I, Miyahara T, Ito T, Migita Y, Nawata H (1995). Giardiasis in pancreas. *The Lancet*.

[25] Chirila M, Panaitescu D, Capraru T (1981). Frequency of *Giardia lamblia* in certain allergic syndromes. *Médecine Interne*.

[26] Hamrick HJ, Moore GW (1983). Giardiasis causing urticaria in a child. *American Journal of Diseases of Children*.

[27] Clyne CA, Eliopoulos GM (1989). Fever and urticaria in acute giardiasis. *Archives of Internal Medicine*.

[28] Almannoni SA, Martín Pupo D, Rodríguez ME (2008). Manifestaciones cutáneas de la giardiasis, sobredimensión de un problema de salud. *Revista Cubana de Medicina Tropical*.

[29] Fonte Galindo L, Almannoni SA, Martín Pupo D, Monzote López A, Sánchez Valdés L, Sayas Berbes M (2010). Conocimientos, percepciones y prácticas en relación con giardiasis: resultados de una encuesta aplicada a gastroenterólogos: Ciudad de La Habana. *Revista Habanera de Ciencias Médicas*.

[30] Iglesias Hernández T, Almannoni SA, Rodríguez ME (2010). Conocimientos, percepciones y prácticas de los dermatólogos en relación con la infección por *Giardia lamblia*. *Revista Cubana de Medicina Tropical*.

[31] World Health Organization (WHO) (2007). *Combating Waterborne Disease at the Household Level/International Network to Promote Household Water Treatment and Safe Storage*.

[32] Karanis P, Kourenti C, Smith H (2007). Waterborne transmission of protozoan parasites: a worldwide review of outbreaks and lessons learnt. *Journal of Water and Health*.

[33] Curtis V, Cairncross S (2003). Effect of washing hands with soap on diarrhoea risk in the community: a systematic review. *The Lancet Infectious Diseases*.

[34] Katz DE, Heisey-Grove D, Beach M, Dicker RC, Matyas BT (2006). Prolonged outbreak of giardiasis with two modes of transmission. *Epidemiology and Infection*.

[35] Ravelomanana T, Rakotomahefa M, Randrianaivo N, Raobijaona SH, Barennes H (2010). Mother’s educational level and children’s illness severity in the emergency unit of Joseph-Raseta-Befelatanana Hospital. What kind of implications?. *Bulletin de la Societe de Pathologie Exotique*.

[36] Nundy S, Gilman RH, Xiao L (2011). Wealth and its associations with enteric parasitic infections in a low-income community in Peru: use of principal component analysis. *American Journal of Tropical Medicine and Hygiene*.

[37] Júlio C, Vilares A, Oleastro M (2012). Prevalence and risk factors for *Giardia* duodenalis infection among children: a case study in Portugal. *Parasites & Vectors*.

[38] Hedayati A, Sadraei J, Ghofranipour F (2008). Relationship between the rate of giardiasis and knowledge and practice of prevention in primary school children in south of Tehran. *Parasitology Research*.

[39] Ross-Degnan D, Soumerai SB, Goel PK (1996). The impact of face-to-face educational outreach on diarrhoea treatment in pharmacies. *Health Policy and Planning*.

